# Active Tube-Shaped Actuator with Embedded Square Rod-Shaped Ionic Polymer-Metal Composites for Robotic-Assisted Manipulation

**DOI:** 10.1155/2018/4031705

**Published:** 2018-03-25

**Authors:** Yanjie Wang, Jiayu Liu, Denglin Zhu, Hualing Chen

**Affiliations:** ^1^School of Mechanical and Electrical Engineering, Hohai University, Changzhou Campus, Changzhou 213022, China; ^2^Department of Mechanical Engineering, Johns Hopkins University, Baltimore, MD 21218, USA; ^3^School of Mechanical Engineering, Xi'an Jiaotong University, Xi'an 710049, China

## Abstract

This paper reports a new technique involving the design, fabrication, and characterization of an ionic polymer-metal composite- (IPMC-) embedded active tube, which can achieve multidegree-of-freedom (MODF) bending motions desirable in many applications, such as a manipulator and an active catheter. However, traditional strip-type IPMC actuators are limited in only being able to generate 1-dimensional bending motion. So, in this paper, we try to develop an approach which involves molding or integrating rod-shaped IPMC actuators into a soft silicone rubber structure to create an active tube. We modified the Nafion solution casting method and developed a complete sequence of a fabrication process for rod-shaped IPMCs with square cross sections and four insulated electrodes on the surface. The silicone gel was cured at a suitable temperature to form a flexible tube using molds fabricated by 3D printing technology. By applying differential voltages to the four electrodes of each IPMC rod-shaped actuator, MDOF bending motions of the active tube can be generated. Experimental results show that such IPMC-embedded tube designs can be used for developing robotic-assisted manipulation.

## 1. Introduction

In recent years, electroactive polymers (EAPs) have been studied in many engineering fields, such as artificial muscles, biomimetic robots, dynamic sensors, and energy harvesters. Ionic polymer-metal composite (IPMC) is considered to be one of the most promising types of EAPs because of its unique advantages including flexibility, large deformation under low driving voltage, and biocompatibility. Application to an electric field causes cations to move toward the cathode with water molecules inside IPMC, thus introducing swelling in the cathode side and shrinking in the anode side, which leads to a bending motion toward the anode [1–3]. Recently, IPMC has been considered for many applications, such as underwater biomimetic robotics [4–9] and energy harvesting devices [10–13].

A traditional strip-type IPMC consists of an ion exchange membrane, typically Nafion (DuPont) membranes with thickness of less than 300 *μ*m, and two thin noble metallic electrodes chemically and physically deposited on the surfaces of the membrane. When an electric field is applied, the actuation mode of a strip IPMC generates 1-dimensional bending toward the anode. An extensive study has been conducted in improving the performance of IPMCs [14], including the development of novel ionic polymer membranes through a polymer blending method and nanocomposite membranes [15–23], the replacement of traditional metallic electrodes by nonmetal electrodes such as carbon nanotube-based electrodes [24, 25], and the optimization of the electrode interface [26], increasing the thickness of Nafion membranes through a solution casting method and a hot pressing technique [27, 28]. Although the performance of IPMCs has been improved, the 1-dimensional bending motion of traditional strip-type IPMCs limits its applications. Many researchers have tried to make IPMC biaxial bending actuators capable of multidegree-of-freedom bending motions. Shahinpoor and Kim proposed the concept of an IPMC biaxial bending actuator in a square rod form [29]. Lee et al. presented platinum-electrode biaxial bending IPMCs based on hot pressing of Nafion films [30]. Cylindrical and tubular platinum-electrode IPMCs based on a hot press process and an extrusion process have also been developed [31–34].

With the development of IPMCs with nontraditional shapes, more promising applications have been proposed and achieved in the biomedical field. Lee et al. presented a helical IPMC actuator for radius control of biomedical active stents [35]. Nguyen et al. developed a flap valve IPMC micropump with a flexible supported diaphragm [36]. Li et al. proposed the application of IPMC-based artificial muscle to the myoelectric hand prosthesis [37]. Yoon et al. reported the development of a low-cost active tip bending system actuated by a strip IPMC actuator at the tip for a scanning fiber endoscope [38]. Moreover, an active catheter or active guide wire systems for biomedical applications based on IPMC actuators have received much attention recently. Strip-type IPMC actuators were attached on the front to serve as the servo actuator to bend the catheter for biomedical applications such as active catheters in bifurcated blood vessels [39–41]. However, the resulting motion is only 1-dimensional bending motion perpendicular to the electrodes of the strip-type IPMC actuator. Rod-shaped or tubular IPMC actuators with outer diameter less than 2 mm have also been successfully developed for biomedical applications in intravascular procedures [14, 31–33, 42]. Rod-shaped and tubular IPMCs with four separated electrodes on the surfaces can bend to multiple directions.

Nowadays, robotic-assisted manipulation has become increasingly popular because of numerous benefits compared with conventional surgical procedures, typically such as minimally invasive surgery (MIS). The most common robotic system currently used in the MIS procedures involves using master-slave systems, such as Da Vinci systems, and the multidegree-of-freedom surgical instrument actuated by cable-driven antagonist actuations [43, 44]. Recently, some new alternative mechanisms have been introduced, including electromagnetic drives and hydraulic/pneumatic actuators [45–47]. Unfortunately, these types of MIS robotics have some limitations of their own, such as bulky size, high stiffness, and structural complexity. We herein propose a novel structure which involves molding or integrating IPMC rod-type actuators into a soft silicone rubber material to create an active tube that can be used to realize multidegree-of-freedom bending motions. Compared with existing technologies in robotic-assisted manipulation, the proposed smart active tube offers some advantages in that it can reduce damage to a minimum due to the softness of the structure. The bending motion of the tube could be controlled to reach the desired location, while surgical instruments could be used during surgery through the hole of the active tube.

**Figure 1:**
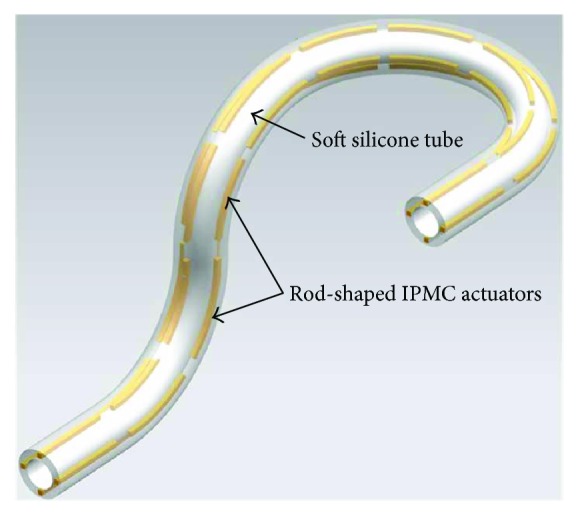
A conceptual appearance of the proposed rod-shaped IPMC-embedded tube actuator.

Figure 1 shows the conceptual design of the proposed active tube-shaped actuator with embedded square rod-shaped IPMC. The main contribution of this work is the design, fabrication, and performance characterization of the newly created prototype of the IPMC-embedded multidegree-of-freedom bending tube structure. The rod-shaped IPMCs were fabricated by Nafion solution casting and deposition of palladium-gold electrodes. The soft silicone elastomer tube structure was cured in molds abricated by 3D printing technology. The fabricated rod-shaped IPMCs with dimension of 25 mm × 1 mm × 1 mm (length × width × thickness) were then integrated with the tube structure. We conducted experiments to investigate the multidegree-of-freedom actuation performance, strain energy density, and efficiency of the IPMC-embedded tube actuator. This paper is organized as follows. First, the fabrication process of the rod-shaped IPMCs is developed. Next, the design and fabrication of the rod-shaped IPMC-embedded active tube actuator are presented. After that, experimental results are reported, and strain energy density and efficiency are evaluated. Concluding remarks are presented in the final section.

## 2. Fabrication Process

### 2.1. Rod-Shaped IPMC Actuator

In this research, palladium-electrode rod-shaped IPMC actuators with square cross section were fabricated in house based on the solution casting method, and gold was electroplated on top of the palladium electrode to enhance the performance of rod-shaped IPMCs. First, we used Nafion PFSA polymer dispersions (DE 520CS, DuPont) to make a 1 mm thick Nafion film from the solution casting method [26, 27]. Ethylene glycol was added into the Nafion solution to avoid forming cracks during the solution casting process. Then, we cut the plate into Nafion rods with square cross section 1 mm × 1 mm. For the detailed description, refer to [48].

After fabricating the Nafion polymer beams with square cross section, an impregnation-reduction process and an electroless palladium plating process were applied to create the metal electrodes on the surface of the fabricated Nafion beams. The preparation process was as follows [49]. First, the Nafion beams were sandblasted to roughen the surface and treated with 3% H_2_O_2_ and 1 mol/l HCl to remove impurities from the beams. Afterwards, the impregnation-reduction process was applied to enable the Nafion beam to absorb enough palladium complex ions Pd(NH_3_)_4_^2+^ which will be reduced to palladium particles that penetrate deep into the Nafion beam. The Nafion beams were immersed in Pd(NH_3_)_4_^2+^ solution (0.1 mol/l) for several hours to allow the Nafion beams to absorb the palladium complex ions sufficiently. The reduction process took place in NaBH_4_ solution (0.85 g/l) as the reduction agent to form electrodes on all six surfaces of the Nafion beam. The impregnation-reduction process was repeated for more than 6 times to increase the penetration of palladium particles into the Nafion beams because Nafion beams are thicker than the typical Nafion membranes. Then, the electroless palladium plating process was performed to further create metal electrodes to reduce the resistance of the surface electrodes. The mixed solution of hydrazine hydrate and Pd(NH_3_)_4_^2+^ was used to deposit more palladium metal on the surface to form a thicker palladium electrode layer. The electroless palladium plating process was repeated 3 times to achieve multiple layers of palladium on all surfaces of the Nafion beams.

The electroplating process was then performed by using gold complexes in order to further improve the electrode conductivity without increasing the stiffness significantly. Over time, the performance degradation of the IPMC actuators can occur with an increase in surface (electrode) resistance due to cracks or voids from high levels of actuation or dehydration. Electroplating of gold on the palladium electrodes can fill the cracks and voids and thus can reduce the surface resistivity and maintain the actuation performance [7]. For newly fabricated rod-shaped IPMCs with palladium electrodes, the electrode resistance on the four surfaces is 12.7 Ω and 1.1 Ω (measured by the two farthest points) before and after the gold electroplating process, separately. In the final step, the four longitudinal edges are insulated by a scalpel and the two ends of the fabricated rod-shaped IPMCs were removed to form 4 separate electrodes on the surface of the Nafion beams. The type of mobile ions inside the IPMCs is sodium ions, with water as solvent.

**Figure 2:**
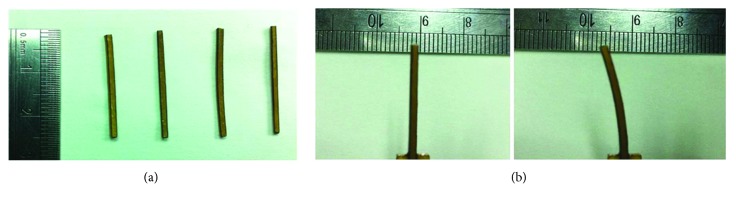
Fabricated rod-shaped IPMC actuators: (a) real optical image; (b) actuation exhibition.

**Figure 3:**
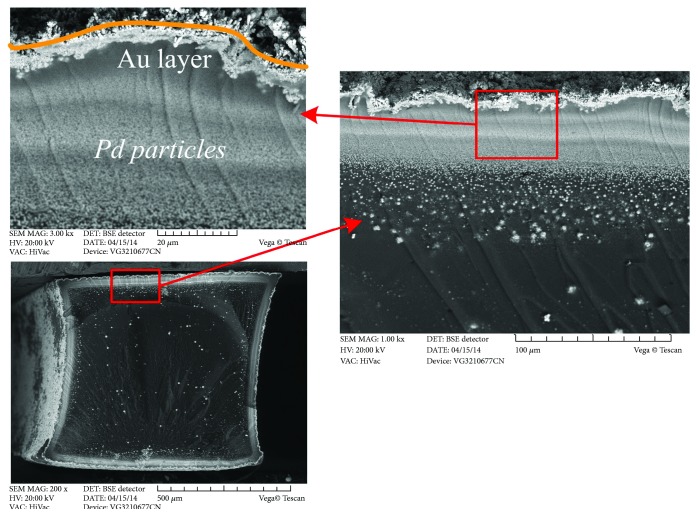
Cross-sectional morphology of rod-shaped IPMCs with palladium-gold electrodes.

Figure 2(a) shows the real optical image of rod-shaped IPMC actuators of 1 mm × 1 mm × 25 mm. It can be seen from Figure 2 that the Au electrode is deposited uniformly on the copolymer surface with the above fabrication process. To confirm the thickness of copolymer and electrodes, Figure 3 shows the SEM images of the cross section. From Figure 3, it is clearly shown that a Pd electrode layer with a thickness of approximately 100 *μ*m was formed. Notably, Pd grains penetrate deep into the Nafion copolymer, and there is no clear interface observed between the Pd grains and Nafion copolymer, which indicates that the electrodes adhere very well with the copolymer. The thick metal electrode layer with excellent adhesion should decrease the surface resistance, increase the current of an IPMC, and enhance the actuation deformation and blocking force.  Figure 2(b) shows the actuation exhibition of a rod-shaped IPMC actuator.

### 2.2. Soft Silicone Sleeve

**Figure 4:**
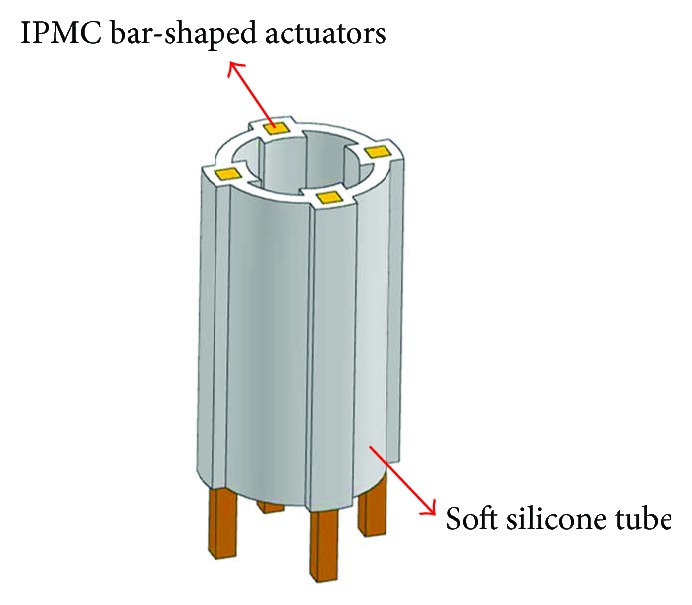
Rod-shaped IPMC-embedded multidegree-of-freedom bending tube actuator.

Figure 4 illustrates the concept of embedding rod-shaped IPMC actuators into a soft tube to create a smart active tube capable of multidegree-of-freedom bending motions. In this research, four rod-shaped IPMC actuators are incorporated into the tube structure to control multidegree-of-freedom bending motions of the overall active tube. Further, more rod-shaped IPMC actuators could be implemented to improve the performance of the active tube actuator. The dimension of the tube structure was outer diameter (10.5 mm) × inner diameter (8.5 mm) × length (20 mm), designed to house four rod-shaped IPMC actuators with square cross section (1 mm × 1 mm × 25 mm). The length of the rod-shaped IPMC actuators is fabricated to be larger than the length of the tube structure to facilitate the connection between the IPMCs and the experimental setup. In order to decrease the stiffness of the silicone tube and improve the actuation performance, the tube is designed to have a final thickness of 0.7 mm over the rod-shaped IPMCs and a thickness of 1 mm between the bumps to house the IPMC actuators.

**Figure 5:**
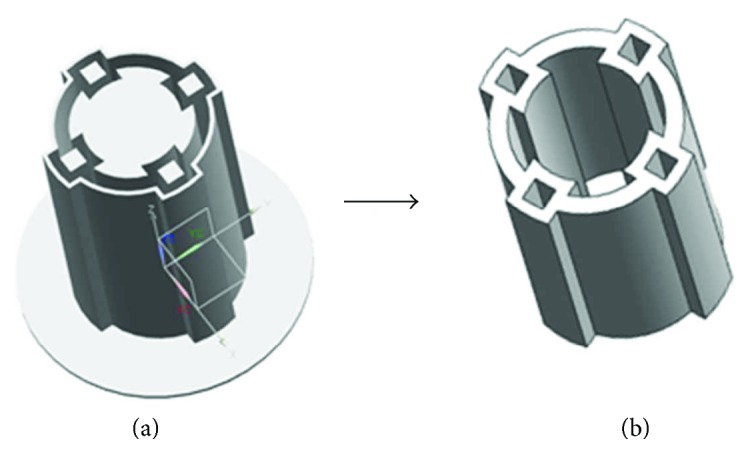
(a) The mold fabricated by 3D printing technology for the silicone rubber tube. (b) Soft silicone tube to house 4 rod-shaped IPMC actuators.

After fabricating rod-shaped IPMC actuators, a mold for the tube structure was fabricated by 3D printing technology using a stereolithography apparatus (SCPS350B, XJRP). The mold was designed to fabricate the silicone rubber tube (outer diameter (10.5 mm), inner diameter (8.5 mm), and (length 20 mm)) with four cavities (1 mm × 1 mm × 20 mm) that can house four rod-shaped IPMCs, as shown in Figure 5. Four bumps with the dimension of 2.4 mm × 2.4 mm at every 90° of the silicone rubber tube were designed to house the rod-shaped IPMCs.

**Figure 6:**
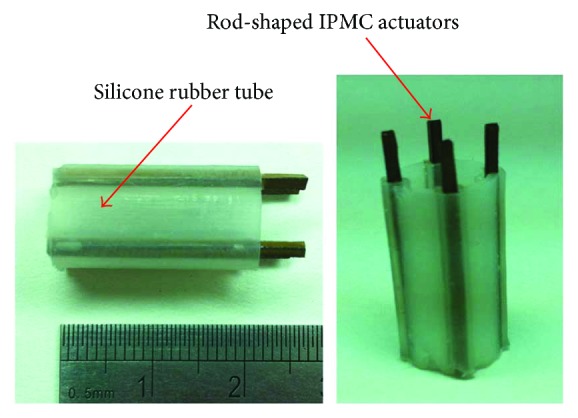
Fabricated rod-shaped IPMC-embedded active tube actuator.

Silicone casting rubber TC5005 A/B-C (BJB Enterprises Inc., USA) was used to fill the mold to make a flexible tube for the rod-shaped IPMCs. The modulus of the silicone elastomer can be controlled through different mixing ratios between the base silicone and the catalyst. In this research, the mixing ratio was chosen to be one part of catalyst per 10 parts of silicone, as indicated by the manufacturer. The mold fabricated by 3D printing technology was first sprayed with a mold release agent. Then, the mixed silicone gel was poured into the mold and allowed to cure at room temperature for 24 h. Next, the mold was removed leaving the silicone rubber tube with 4 cavities to insert rod-shaped IPMCs. Four rod-shaped IPMC actuators were then integrated into the silicone rubber tube to complete the IPMC-embedded active control tube (Figure 6).

### 2.3. Motion Analysis of Rod IPMC and IPMC-Embedded Active Tube

**Figure 7:**
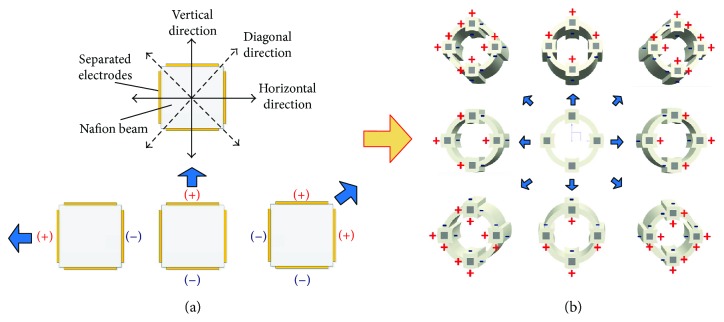
Actuation modes of (a) rod-shaped IPMC and (b) IPMC-embedded tube in the vertical/horizontal directions and diagonal directions.

The applied voltages to the four electrode surfaces of a rod-shaped IPMC could be independently controlled. Thus, a rod-shaped IPMC actuator is capable of bending to eight directions including the vertical/horizontal directions and the diagonal directions (Figure 7(a)). To actuate a rod-shaped IPMC in the vertical/horizontal directions, one positive signal was applied to the corresponding electrode. Positive signals were simultaneously applied to adjacent electrodes to actuate rod-shaped IPMC actuators in the diagonal directions. Thus, by controlling the voltages applied to the four electrodes of each rod-shaped IPMC actuator, the bending motions of four inserted rod-shaped IPMC actuators can be independently controlled, causing the multidegree-of-freedom bending motions of the overall active tube structure (Figure 7(b)).

### 2.4. Experimental Setup

**Figure 8:**
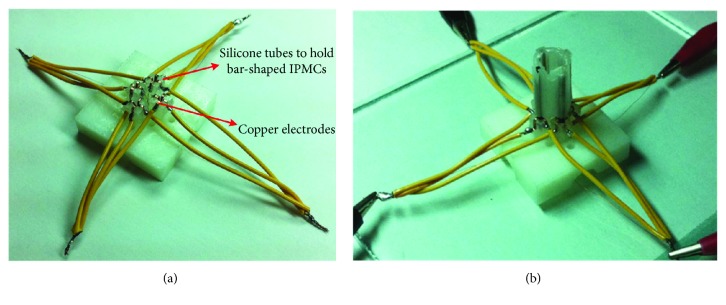
(a) Experimental setup for the IPMC-embedded tube actuator. (b) The assembly ready for measurement. Due to space constraints, fine copper wire is used for the fourth conductive wires inside the active tube.

In order to independently apply electric signals to the four electrodes of each rod-shaped IPMC actuator, a custom clamp is created to maintain good electrical contact between the metallized surfaces of the IPMCs and the electrodes of the clamp. As shown in Figure 8, four silicone tubes were inserted into the base fabricated by 3D printing technology. Sixteen pieces of trimmed copper sheets were attached to the silicone tubes as the electrical pads to apply electric signals to the four electrodes of each rod-shaped IPMC actuator. Sixteen wires are individually soldered to the copper sheets for transmitting electrical signals to the device. The active tube actuator embedded with four rod-shaped IPMCs was then inserted into the clamp. A PC equipped with LabVIEW software and a power supply applies the necessary controlled activation signals to the overall sixteen electrodes of four rod-shaped IPMCs through copper sheets. The bending deflection and blocking force of the fabricated IPMC-embedded tube actuator were measured at the tip using a laser displacement sensor (Model Keyence LK-G80) and a force sensor (Model Keyence GSO-10).

## 3. Results and Discussions

### 3.1. Tip Deformation and Current Characterization of the Active Tube Actuator

The bending deformation of the active tube actuator in the vertical/horizontal direction and diagonal direction was measured at the tip of the tube using a laser displacement sensor. The current of rod-shaped IPMCs was simultaneously measured and recorded. The four rod-shaped IPMCs were actuated at 0.2 Hz with both sine and square wave input signals of 0.5 V–2.0 V with the interval of 0.5 V, respectively.

**Figure 9:**
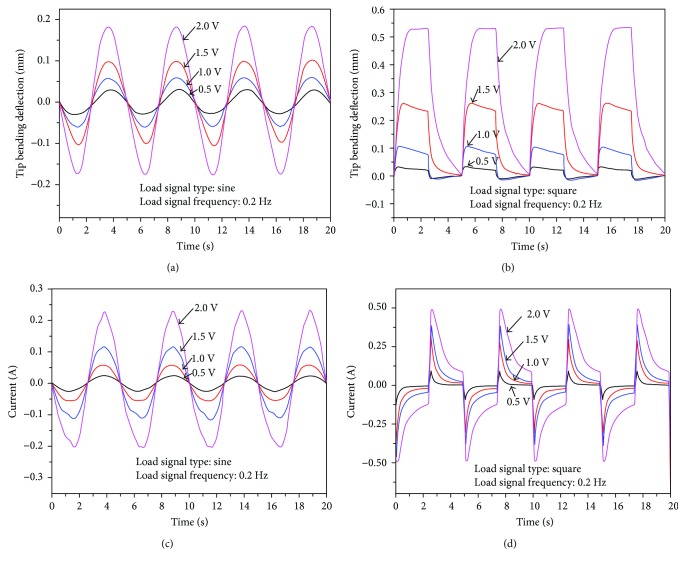
Experimental results in the vertical/horizontal directions: (a) tip deformation under sine input signals, (b) tip deformation under square input signals, (c) current responses under sine input signals, and (d) current responses under square input signals.

First, the active tube bending deformation in the vertical/horizontal direction was measured. In order to actuate the IPMC-embedded tube actuator in the vertical/horizontal directions, the same input signal with the same phase was applied to the corresponding electrodes of all four rod-shaped IPMCs (see Figure 7(b)). The tip bending responses of the active tube actuator when sine and square wave input signals of 0.5 V, 1.0 V, 1.5 V, and 2.0 V at 0.2 Hz were applied are shown in Figure 9. As the driving voltage increases, the bending deformation of the active tube actuator increases. The experimental data reveals that the tip bending deformation under square input signal is larger compared to that under sine wave input signal. This is expected as the largest tip bending deformation in the vertical/horizontal direction is 0.53 mm under 2.0 V square wave input at 0.2 Hz. The responses exhibit a good repeatability under periodic electrical inputs. Figures 9(c) and 9(d) show the current responses of the active tube actuator.

**Figure 10:**
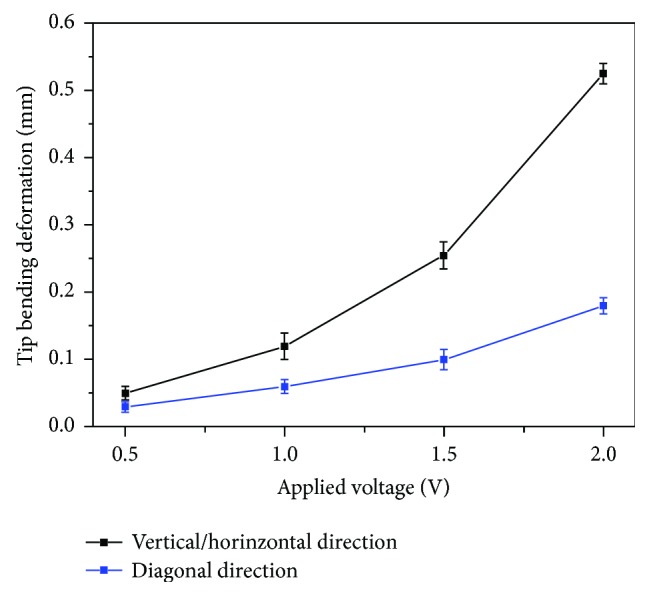
Tip bending deformation in the vertical/horizontal directions under 0.2 Hz actuation.

From the relation between tip bending deformation in the vertical/horizontal directions and applied voltages (Figure 10), we can also observe that the bending deformation of the active tube actuator does not increase linearly with the driving voltage. This is because the actuation of IPMCs is induced by the migration of cations with water molecules from anode to cathode inside the material. When the applied voltage increases, much more cations can move along with water molecules inside the IPMCs, thus generating a larger bending deformation of the entire tube structure. In fact, a larger increment of bending deformation can be achieved each time we increase the driving voltage by an interval of 0.5 V from 0.5 V to 2.0 V. Much larger bending deformations can be generated by applying higher voltages. However, electrolysis due to high driving voltages should be taken into account to ensure stable actuation performance.

**Figure 11:**
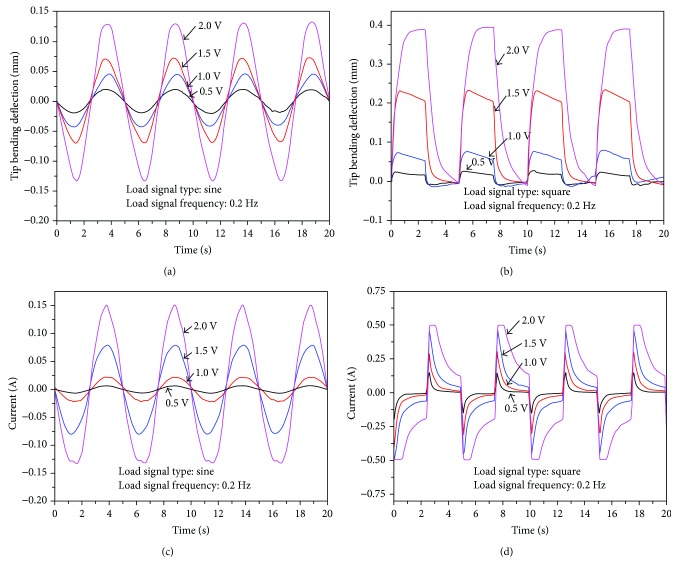
Experimental results in the diagonal directions: (a) tip deformation under sine input signals, (b) tip deformation under square input signals, (c) current responses under sine input signals, and (d) current responses under square input signals.

Next, the bending deformation of the active tube actuator in the diagonal direction is generated by simultaneously applying the same input to the corresponding adjacent electrodes of each rod-shaped IPMC actuator (Figure 7(b)). The same levels of the above sine and square wave signals were applied to actuate the four rod-shaped IPMCs.  Figure 11 illustrates the tip bending deformation in the diagonal directions of the active tube actuator under different input signals. The same trend was observed that utilizing a square input signal generates a larger bending response compared to the sine input signal. The tip bending deformation of the tube actuator in the diagonal direction was smaller than that in the vertical/horizontal direction under the same input signal. The largest tip bending deformation in the diagonal direction is approximately 0.4 mm under 2.0 V square wave input at 0.2 Hz.

It should be noted that, although the tip bending deformation of the tube actuator in the diagonal direction was smaller than that in the vertical/horizontal direction under the square input signal, the current responses of IPMCs show the opposite trend. By comparing Figure 9(c) with Figure 11(c), we find that the actuation current in the diagonal direction was larger than that in the vertical/horizontal direction under the square input signal. This is due to the fact that in the diagonal direction, the electric field strength between the adjacent anode and cathode is higher than that between two opposite outer electrodes of rod-shaped IPMCs in the vertical/horizontal direction. This in turn causes higher charge density near the electrodes and therefore higher actuation current. However, the bending stiffness of rod-shaped IPMCs in the diagonal direction is larger than that in the vertical/horizontal direction. Thus, although the actuation current in the diagonal direction is larger, the bending deformation of the tube structure in the diagonal direction is smaller than that in the vertical/horizontal direction. In addition, for Figure 11(d), the current data under square input signal of 2.0 V has overrange. The peak current is due to the charging process of IPMC. Since the acquisition circuit of the maximum current we designed is 0.5 A, the data above 0.5 A cannot be obtained. But from the present data, the current responses at different voltages have clearly shown an approximately linear trend.

### 3.2. Tip Blocking Force Characterization of the Tube Actuator

**Figure 12:**
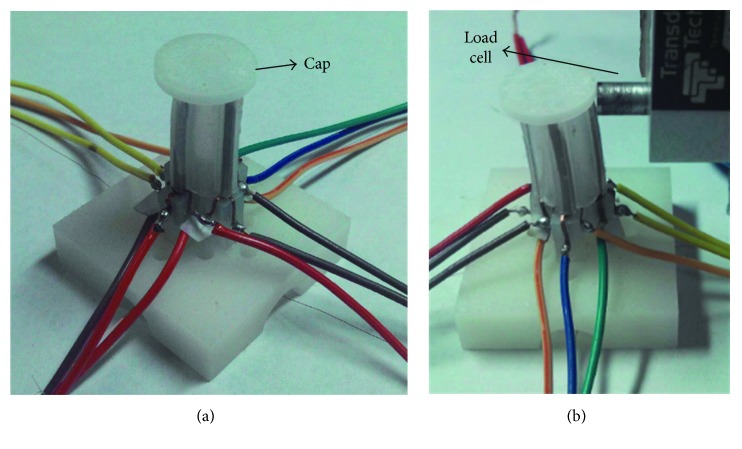
Experimental setup for measuring tip bending force of the IPMC-embedded tube actuator.

A lightweight cap fabricated by 3D printing technology was put on top of the active tube actuator so that the blocking force of the entire tube actuator could be measured and the actuation performance of the tube actuator would not be affected (Figure 12). The bending force of the active tube in the vertical/horizontal direction and the diagonal direction was measured at the tip using a load cell. The same actuation scheme as aforementioned was used to actuate the tube actuator in the vertical/horizontal directions and the diagonal directions.

**Figure 13:**
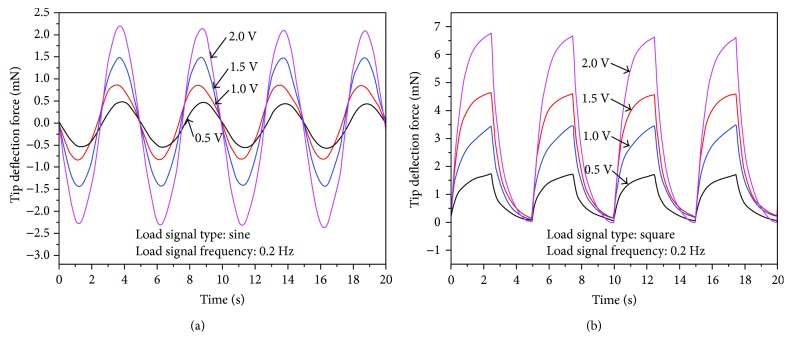
Measured tip bending force in the vertical/horizontal directions under (a) sine input signals and (b) square input signals.

For the tip bending force of the tube actuator in the vertical/horizontal directions, 0.2 Hz 0.5 V–2.0 V sine and square wave input signals were applied to the four rod-shaped IPMCs (Figure 13). The tip bending force of the active tube actuator increases with the driving voltages, and the tip bending force under square input signals is larger than that under sine input signal. The largest tip bending force in the vertical/horizontal directions is 6.9 mN under 0.2 Hz 2.0 V square wave input signal.

**Figure 14:**
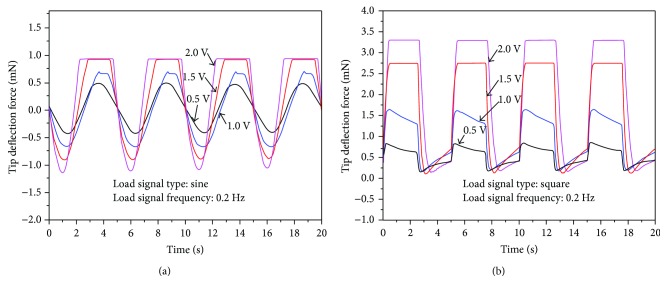
Measured tip bending force in the diagonal direction under (a) sine input signals and (b) square input signals.

Figure 14 shows the tip bending forces in the diagonal direction for the tube actuator under 0.2 Hz 0.5 V–2.0 V sine and square input signals. From Figure 14, tip force data of cases 1.5 V and 2.0 V both have overrange at the diagonal direction. This is related to our initial setup. It is well known that the force response of IPMC is hard to measure. In order to measure the force response of the active tube, we design a plastic cap to cover the top of the tube. When the test begins, that cap gives the force sensor a prestress (Figure 12(a)). With the application of alternating voltage, the tube with the cap touches or leaves the force sensor inevitably. When the cap leaves the force sensor, the force response cannot be obtained as shown in Figure 14. If the prestress is too large, force response data will be inaccurate. We used a smaller prestress in the experiments so that overrange is inevitable. Even so, it is conceivable that all data of four cases show a sinusoidal form. The offset and irregular phenomenon is due to the effect of electrodes in the diagonal direction and the silicone tube. The tip bending force of the tube actuator in the diagonal direction was smaller than that in the vertical/horizontal direction under the same input signal. Additionally, for the sine driving signals with amplitudes higher than 1.0 V, the maximum tip bending force in the diagonal direction was not reached (Figure 14(a)). This is because the cap on top of the tube actuator was initially pushed in touch with the load cell before measurement (Figure 12). When the tube bends toward the load cell, the tip bending force of the active tube could be accurately measured (negative force values). When the tube bends away from the load cell, the tip bending force could not be measured after the tip leaves the load cell (positive force values). Therefore, we can observe that for driving signals with amplitudes higher than 1.0 V, the negative force values have reached the maximum, but the positive force values have not reached the maximum. The same phenomenon could be observed for tip bending force in the diagonal direction under square wave signals in Figure 14(b).

**Figure 15:**
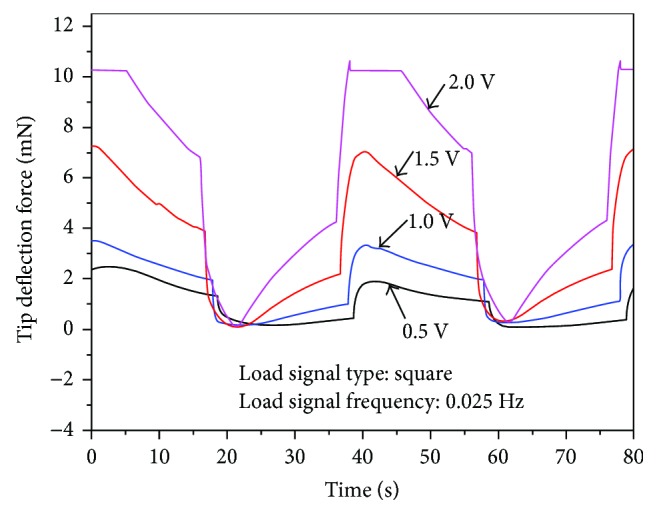
Measured tip bending force in the vertical/horizontal directions under 0.025 Hz square input signals.

Another observation is that the tip bending forces in the vertical/horizontal direction under 0.2 Hz 0.5 V–2.0 V square wave input signals have not reached the maximum values that could be achieved with such driving voltages (Figure 13(b)). The tip bending forces were still increasing and have not reached their maximum values when the driving voltage started to decrease and the tube started to bend toward the opposite direction. Thus, the tip bending forces of the tube actuator in the vertical/horizontal direction were measured under 0.025 Hz 0.5 V–2.0 V square wave input signals (Figure 15). The resulting tip bending forces in the vertical/horizontal direction reach the maximum values, and the largest tip bending force reaches up to 10.4 mN under 0.025 Hz 2.0 V square wave signal. The same problem arises that the force values could not reach the maximum for the 0.025 Hz 2.0 V square input due to the initial pressure between the cap and the load cell. The present result also shows that the tip bending force of the IPMC-embedded tube actuator was not able to maintain the maximum value and gradually declined. These experimental data suggest that the relaxation back phenomenon that appears in flat-type IPMC actuators can occur in the step responses of the rod-shaped IPMC actuators.

## 4. Conclusions

This paper explored the approach to embed rod-shaped IPMC actuators into a soft tube structure to create an active tube capable of multidegree-of-freedom bending motions. The Nafion solution casting method was modified, and a complete sequence of the fabrication process was developed for rod-shaped IPMCs with square cross sections. Four rod-shaped IPMCs were incorporated into the soft tube fabricated based on 3D printing technology to create the active tube actuator. For the IPMC-embedded tube with dimensions of OD (10.5 mm) × ID (8.5 mm) × L (20 mm), the measured maximum tip bending deformation in the vertical/horizontal direction and the diagonal direction was approximately 0.53 mm and 0.4 mm under 0.2 Hz 2.0 V square wave actuation, and the corresponding tip bending force could reach up to 10.4 mN and 3.38 mN. These results suggest that such active tube structures have a potential to be used for minimally invasive surgery applications.
